# USP7 Promotes deubiquitination and stabilization of MyD88 to enhance immune responses

**DOI:** 10.3389/fimmu.2022.900243

**Published:** 2022-08-12

**Authors:** Na Zhang, Fei Wang, Gaomeng Zhang, Qi Zhang, Yuhong Liu, Qiao Wang, Mohamed Shafey Elsharkawy, Maiqing Zheng, Jie Wen, Guiping Zhao, Qinghe Li

**Affiliations:** ^1^ Key Laboratory of Animal (Poultry) Genetics Breeding and Reproduction, Ministry of Agriculture and Rural Affairs, Institute of Animal Sciences, Chinese Academy of Agricultural Sciences, Beijing, China; ^2^ Key Laboratory of Animal Genetics, Breeding and Reproduction of Shaanxi Province, College of Animal Science and Technology, Northwest Agriculture and Forestry (A&F) University, Yangling, China; ^3^ Animal Production Department, National Research Centre, Cairo, Egypt; ^4^ Hainan Yazhou Bay Seed Laboratory, Sanya, China

**Keywords:** USP7, MyD88, deubiquitinase, cytokines, innate immunity

## Abstract

Toll-like receptors (TLRs) are involved in the sensing of pathogen-associated molecular patterns (PAMPs) such as lipopolysaccharide (LPS), flagellin, unmethylated double-stranded DNA (CpG), single-stranded RNA (ssRNA) and lipoproteins. Myeloid differentiation primary response protein 88 (MyD88) is a canonical adaptor for the Toll-like receptor family which has crucial roles in host defense against infection by microbial pathogens. The dysregulation of MyD88 may also induce autoimmune diseases. Here, we demonstrate that the deubiquitinase USP7 interacts with MyD88 in chicken, with knockdown or overexpression of USP7 leading to the regulation of MyD88 protein in a positive manner. Consequently, USP7 positively regulates the expression of proinflammatory factors upon LPS challenge. Furthermore, we observed USP7-deficient mice to be more susceptible to infection by Salmonella typhimurium. Collectively, our findings demonstrate MyD88 as a bona fide substrate of USP7 and uncover a mechanism by which USP7 regulates innate immune signaling.

## Introduction

The host innate immune system composes the first line of defense against pathogenic microorganisms. The innate immune system relies on pattern-recognition receptors (PRRs) to detect pathogen-associated molecular patterns (PAMPs) in the invasive pathogens (1, 2). Toll-like receptors are the most important kind of transmembrane PRRs, recognizing bacteria, double-stranded and single-stranded RNA viruses, and flagellins (3). MyD88 is the canonical adaptor that links TLR receptors with the downstream IRAK family members; most TLR receptors rely on binding of MyD88 with their cytoplasmic portions to convert infectious signals and activate downstream innate immune pathways (4). Activation of TLR signaling leads to the production of inflammatory cytokines, type I interferons, and chemokines, and the induction of immune responses to eliminate the invading pathogens.

Overall, signal transduction in immune cells is subject to stringent regulation, including through the post-translational modification of proteins critical to immune signaling pathways. Ubiquitination is a reversible post-translational modification in which ubiquitin is conjugated to lysine residues in target proteins, and is widely involved in the regulation of diverse biological processes. Ubiquitination reactions are catalyzed by the sequential action of three classes of enzymes: E1 ubiquitin-activating enzymes, E2 ubiquitin-conjugating enzymes, and E3 ubiquitin ligases (5), of which the last determine substrate specificity; more than 600 E3 ligases have been identified in mammals. Conjugated ubiquitin chains can later be removed by a large number of ubiquitin-specific proteases, which are termed deubiquitinases (DUBs).

Many members of the innate immune pathway, such as MyD88, TRAF6, TBK1 and RIP1, are subjected to E3 ligase-mediated ubiquitination. MyD88 is a central adaptor of the TLR-NF-κB signaling that converts signals from TLR receptors to activation of downstream pathways. Ubiquitination and deubiquitination of MyD88 have crucial impacts on TLR signaling activity and hence on innate immune responses (6, 7). Several E3 ligases have been identified as regulators of MyD88 protein abundance and TLR signaling, including Nrdp1, Smurf, Cbl-b, and SPOP (6–10). Meanwhile, the deubiquitinases CYLD and OTUD4 have been revealed as able to cleave K63-linked polyubiquitination of MyD88 and thus to deactivate TLR-mediated NF-κB signaling (11, 12). However, no K48-linked MyD88 deubiquitinase has yet been found, and how this kind of degradation-promotive polyubiquitination is removed from MyD88 remains poorly defined.

## Methods

### Ethics statement

Animal care and use protocols were performed in accordance with the regulations in the Guide for the Care and Use of Laboratory Animals issued by the Ministry of Science and Technology of the People’s Republic of China. The animal experiments were approved by the Animal Ethics Committee of the Institute of Animal Sciences, Chinese Academy of Agricultural Sciences (Approval Number: IAS2018-8).

### Cell culture and transfection

The chicken fibroblast cell line DF1 was maintained in Dulbecco’s Modified Eagle’s Medium supplemented with 10% fetal bovine serum (FBS, Gibco), 100 μg/mL streptomycin, and 100 units/mL penicillin at 37°C. The chicken macrophage cell line HD11 was cultured in RPMI 1640 medium supplemented with 10% FBS, 10 mM HEPES, 2 mM glutamine, 1 mM sodium pyruvate, 0.1 mM non-essential amino acids, 100 units/mL penicillin, 100 μg/mL streptomycin, and 5×10^-5^ M 2-mercaptoethanol. All cells were maintained in a humidified incubator at 37°C with 5% CO_2_. For plasmid transfections, the Lipofectamine 3000 transfection reagent (Life Technologies) was used according to the manufacturer’s instructions. For siRNA transfections, the transIT-2020 Transfection Reagent (Mirus Bio) was used.

### Antibodies and reagents

Antibodies against FLAG and MYC (dilution 1:3,000) were purchased from Abmart. Monoclonal antibody against USP7 was obtained from Santa Cruz Biotechnology (1:1,000). Rabbit anti-MyD88 was sourced from Cell Signaling Technology (1:2000). Mouse anti-β-actin was obtained from Proteintech (1:3000). Goat anti-mouse and goat anti-rabbit secondary HRP-conjugated antibodies were purchased from Abcam. LPS (*Escherichia coli* serotype O55:B4) was purchased from Sigma-Aldrich.

### Plasmids

*USP7* and *MyD88* sequences were amplified by a high-fidelity DNA polymerase (Takara) using cDNA from DF1 cells. Purified full-length *USP7* and *MyD88* were cloned into the pcDNA3.1 vector with respective MYC and 3×FLAG tags at the C–terminal end. All constructs were confirmed by sequencing.

### RNA interference

All siRNAs were designed and synthesized by Gene-Pharma. Synthesized siRNAs were diluted with RNase-free water. HD11 cells at 90% confluence were transfected with 150 nM siRNA specific for the chicken *USP7* gene. The siRNA sequences were:

siRNA#1: 5’- GCCGACACCAAUAUCUCAATT -3’;
siRNA#2: 5’- GCAGCCUGUUACGGACCAUTT -3’;
siRNA#3: 5’- GCGGUCAGAGGCUACCUUUTT -3’;
siNC: 5’-UUCUCCGAACGUGUCACGUTT -3’

### Protein co-immunoprecipitation and immunoblot analysis

Cells were collected 24 h after transfection and lysed in RIPA buffer (50 mM Tris–HCl, pH 7.4, 150 mM NaCl, 0.25% deoxycholic acid, 1 mM EDTA, 1% NP-40, and 0.5% SDS supplemented with protease inhibitor from Roche). Whole cell lysates were precleared with protein A/G beads (Abmart) and incubated with anti-FLAG beads or anti-MYC beads (Abmart) overnight at 4°C. After extensive washing, the beads were boiled in sample loading buffer for 10 min to elute precipitated proteins for immunoblots.

Transfected cells were likewise harvested for immunoblot analyses. Protein lysates or immunoprecipitates were separated on SDS-PAGE gels by electrophoresis and transferred to polyvinylidene fluoride membranes. The membranes were then blocked with 5% skim milk (BD-Pharmingen) and incubated with antibodies for proteins of interest. Blots were developed with Immobilon Western Chemiluminescent HRP Substrate (Millipore) according to the manufacturer’s instructions.

### RNA extraction and quantitative real-time PCR

Total RNA was isolated from cells using TRIzol (Invitrogen) according to the manufacturer’s instructions, then transcribed into cDNA using PrimeScript First Strand cDNA Synthesis Kits (TaKaRa). Transcript abundance of genes of interest was determined by real-time PCR, with primers as follows:

β-actin: sense 5′- GAGAAATTGTGCGTGACATCA -3′,
antisense 5′- CCTGAACCTCTCATTGCCA -3′;
USP7: sense 5′- GGAAGCTGGAGATGCAGAT -3′,
antisense 5′- GCCAACTTGTATCATCTTCC -3′;
MyD88: sense 5′- TGGAGGAGGACTGCAAGAAGT -3′,
antisense 5′- GCCCATCAGCTCTGAAGTCTT -3′;
IL1β: sense 5′- GCATCAAGGGCTACAAGCTCT -3′,
antisense 5′- CCAGGCGGTAGAAGATGAAG -3′;
IL8: sense 5′- TCCTCCTGGTTTCAGCTGCT -3′,
antisense 5′- GTGGATGAACTTAGAATGAGTG -3′.

### P5091 treated mice

8-weeks old mice were tail intravenous injected with 10 mg/kg bodyweight P5091 every three days for 6 times total to inhibit the activity of USP7. Seven days after the last injection, mice were placed in a pathogen free isolator and challenged with *Salmonella typhimurium* (5*10^8^ CFU).

### Statistics and data analysis

Each experiment in this study was repeated at least three times. Fold changes in mRNA levels were compared using one-way ANOVA. Data are expressed as means ± SD.

## Results

### ChUSP7 promotes protein stability of chMyD88

**Figure 1:**
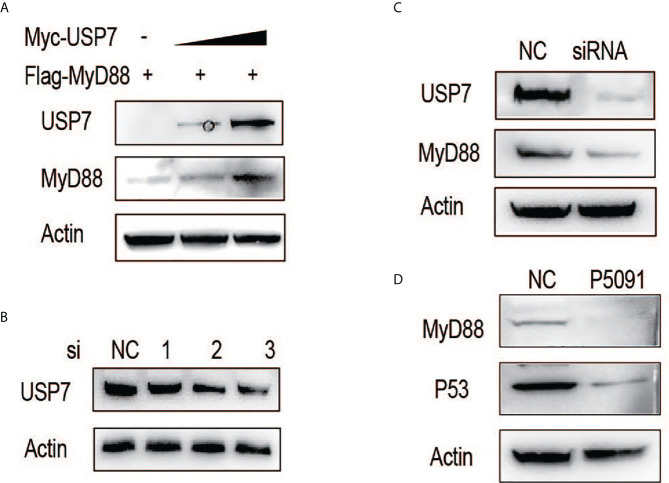
ChUSP7 promotes protein stability of chMyD88. **(A)** Chicken DF1 cells were transfected with increasing doses of Myc-tagged chUSP7, and expression of the indicated proteins was analyzed by immunoblot. **(B)** Knockdown efficiency of different siRNAs designed to target chUSP7. The protein levels of chUSP7 were examined by western blot. **(C)** Immunoblot analysis of endogenous chMyD88 in cells transfected with siRNA against chUSP7. **(D)** Immunoblot analysis of MyD88 in P5091 treated CHO cells. P53 was a previously identified target inhibited by P5091 to show the effect of USP7.

We first examined the effect of chUSP7 on the protein abundance of chMyD88. Our results showed that exogenously-introduced chUSP7 efficiently increased chMyD88 protein level in a dose-dependent manner (**Figure 1A**). We also investigated the effect of *chUSP7* loss on chMyD88 by silencing its expression using short interfering RNA (siRNA). We tested three pairs of siRNAs designed to interfere with *chUSP7* expression, of which siRNA-3 resulted in a significant 50% decrease of chUSP7 (**Figure 1B** and **Supplementary Figure S1**). Accordingly, siRNA-3 was used in all *chUSP7* knockdown experiments hereafter. Knockdown of endogenous *chUSP7* in the presence of the translation inhibitor cycloheximide led to an increase in chMyD88 abundance (**Figure 1C**). However, the mRNA abundance of *chMyD88* remained unchanged, indicating that chUSP7 regulates chMyD88 at the post-translational level rather than the transcriptional level (**Supplementary Figure S2**). To test conservation of the function of USP7 in regulating MyD88, we treated Chinese hamster ovary cells (CHO cells) with P5091 which was a well-known USP7 inhibitor (13). As expected, the protein level of MyD88 significantly decreased in P5091 treated mouse cells (**Figure 1D**), suggesting a highly conserved regulatory role of USP7 on MyD88.

### ChUSP7 interacts and colocalizes with chMyD88 and promotes its K48-linked deubiquitination

**Figure 2:**
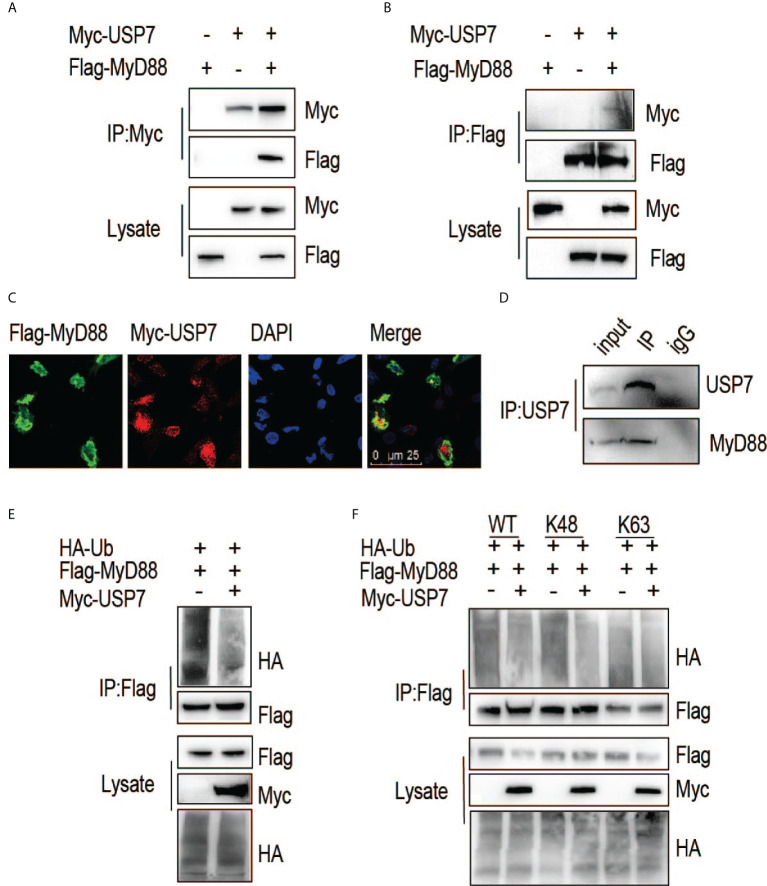
ChUSP7 interacts with and promotes K48 linked deubiquitination of chMyD88. Chicken DF1 cells were transfected with the indicated plasmids. Interaction between chMyD88 and chUSP7 was detected by immunoprecipitation using anti-Myc **(A)** or anti-FLAG **(B)** antibody, followed by immunoblotting with indicated antibodies. **(C)** Immunofluorescence analysis of chMyD88 and chUSP7. Myc-tagged chUSP7 and FLAG-tagged chMyD88 were transfected into chicken DF1 cells, after which the cells were fixed and incubated with anti-Myc and anti-FLAG antibodies. Nuclei were stained with DAPI. Confocal microscopy was used to detect the colocalization of chMyD88 and chUSP7. **(D)** Co-immunoprecipitation of endogenous USP7 and MyD88 in mouse CHO cells. Cell lysates were immunoprecipitated by anti-USP7 or control IgG antibody, followed by immunoblot with indicated antibodies. **(E, F)** Immunoblot analysis of the ubiquitination level of immunoprecipitated chMyD88 in chicken DF1 cells transfected with the indicated plasmids. Immunoprecipitation was carried out with anti-FLAG antibody, and immunoblots with the indicated antibodies.

We next asked if chUSP7 interacts and colocalizes with chMyD88. An association between chMyD88 and chUSP7 was confirmed by constructing expression vectors for the encoding genes and transfecting them into chicken embryonic fibroblast (DF1) cells. We then performed immunoprecipitation analysis and demonstrated that chUSP7 interacts with chMyD88 (**Figure 2A**). We also performed immunoprecipitation with an antibody against chMyD88 and found that chMyD88 and chUSP7 each physically associate with the other (**Figure 2B**). To further confirm this interaction, we carried out immunofluorescence staining analysis, which revealed that chUSP7 colocalizes with chMyD88 (**Figure 2C**). Taken together, these data suggest that chUSP7 interacts and colocalizes with chMyD88. Moreover, we also tested the interaction of USP7 with MyD88 in mice, we observed that endogenous MyD88 could be co-immunoprecipitated with USP7 in mouse CHO cells (**Figure 2D**).

To further substantiate the interaction of these proteins, we next investigated whether chUSP7 could eliminate polyubiquitin chains from chMyD88. As expected, overexpression of *chUSP7* in chicken DF1 cells led to significant deubiquitination of chMyD88 (**Figure 2E**). Notably, different types of polyubiquitin chains result in different fates for the target protein; K48-linked polyubiquitin chains are typically associated with proteasome-dependent degradation and regulation of target protein abundance. As we observed chUSP7 to positively regulate the protein abundance of chMyD88, we transfected DF1 cells with vectors expressing HA-tagged K48- or K63-linked ubiquitin, and found that overexpression of *chUSP7* efficiently decreased K48-linked rather than K63-linked ubiquitination of chMyD88 (**Figure 2F**).

### ChUSP7 positively regulates proinflammatory cytokine production in chicken macrophages

**Figure 3:**
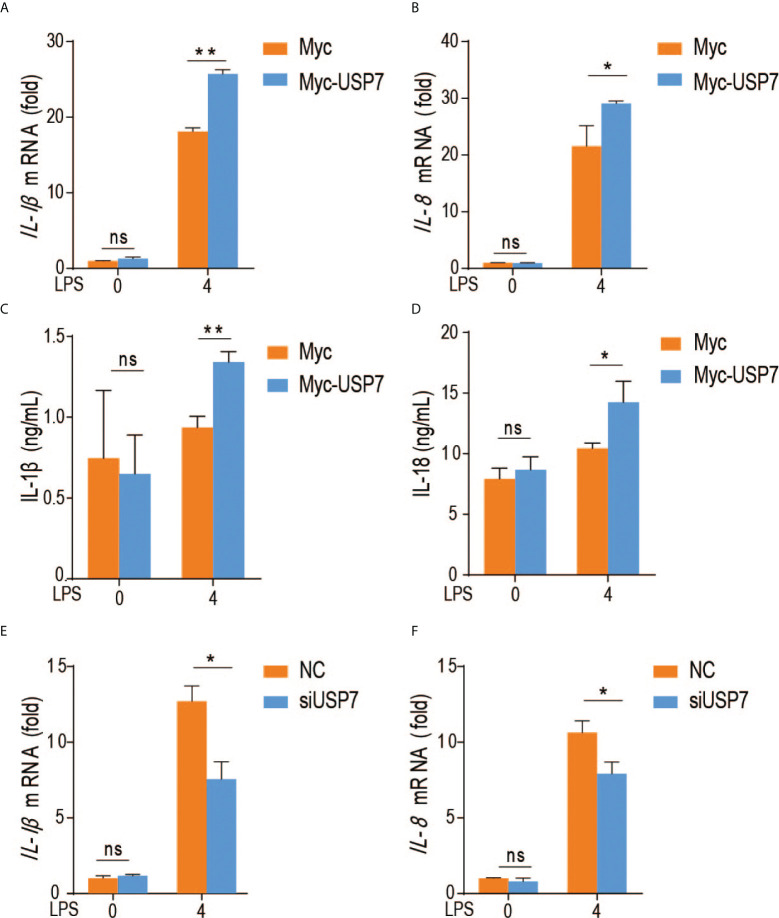
ChUSP7 affects the expression of immune factors in HD11. Abundance of IL-1β and IL-8 mRNA **(A, B)** and protein levels **(C, D)** in chicken HD11 macrophages and cell supernatants with overexpressed **(E, F)** chUSP7 after stimulation with LPS for 4 h. IL-1β and IL-8 mRNA abundances in chicken macrophages transfected with siRNA for chUSP7 after stimulation with LPS for 4 h. *p < 0.05, **p < 0.01; error bars reflect ± SD. ns, Not Statistically Significant.

To evaluate the effect of chUSP7 on production of proinflammatory factors by chicken macrophages, we overexpressed and knocked down *chUSP7* in chicken macrophage cells (HD11), treated those cells with the TLR4 agonist lipopolysaccharide (LPS), and measured the expression of *IL-1β* and *IL-8* as indicators of proinflammatory responses. qRT-PCR assays revealed that overexpression of *chUSP7* significantly decreased the levels of IL-1β and IL-8 in LPS-challenged macrophages (**Figures 3A** **–D**). Correspondingly, knockdown of *chUSP7* in challenged macrophages resulted in greater expression of *IL-1β* and *IL-8* (**Figures 3E** **, F**). These results demonstrate that chUSP7 enhances the MyD88-mediated proinflammatory response in LPS-treated cells.

### Inactivation of USP7 deubiquitination activity leads to attenuated innate immune responses

**Figure 4:**
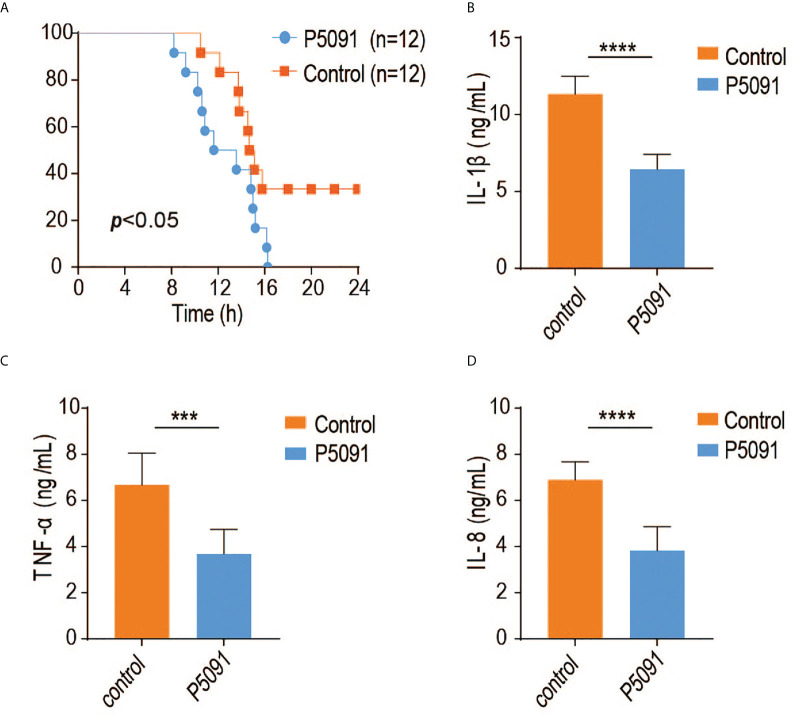
Inactivation of USP7 deubiquitination activity leads to attenuated innate immune responses. **(A)** P5091-treated mice were challenged with Salmonella typhimurium (5×10^8^ CFU) and their survival rates monitored for 24 h (n = 12 each group; statistical analysis: Mantel–Cox test). **(B–D)** ELISA of IL-1β, IFN-α, and IL-6 in the serum of P5091-treated mice challenged with *Salmonella typhimurium* for 6 h (n = 7 per group). ***p<0.001; ****p<0.0001.

To elucidate the *in vivo* function of USP7, we generated a mouse model of USP7 deficiency using the small molecule P5091, previously identified as a specific inhibitor of USP7 deubiquitinase activity. In the current study, mice were first tail intravenous injected with P5091 for six times over 3 weeks to inactivate USP7, then challenged intraperitoneally with *Salmonella typhimurium* and their survival rates were monitored. As expected, USP7-inactivated mice were more susceptible to infection with *Salmonella typhimurium* (**Figure 4A**). Since the USP7-mediated deubiquitination of MyD88 results in greater stability of MyD88 protein, and an uncontrolled innate immune response always results in greater susceptibility to infection, we speculated that P5091-treated mice would also exhibit less severe inflammatory responses. To address this hypothesis, we examined the expression of proinflammatory cytokines and chemokines in the mouse spleen. Treated mice demonstrated lower expression of genes encoding proinflammatory cytokines and chemokines, such as *IL-1β*, *IL-6*, and *TNF-α* (**Figures 4B** **–D**), suggesting that inactivation of USP7 deubiquitination activity leads to attenuated innate immune responses.

## Discussion

MyD88 functions as a central hub in innate immune responses, receiving signals from several receptors that sense various types of pathogen-associated molecular patterns, located either at the plasma membrane or in endosomes. Activation of downstream NF-κB signaling through MyD88 results in the production of pro- or anti-inflammatory cytokines as well as type I IFNs, which antagonize the infection of bacterias or single-stranded RNA virus. As cellular level of MyD88 is a critical factor in maintaining immune homeostasis, MyD88 stability is under strict regulation. The protein is subject to multiple types of post-translational modification, including phosphorylation, K48- and K63-linked ubiquitination (6, 7). Several E3 ligases have been identified to associate with and ubiquitinate MyD88, leading to its proteasomal-dependent degradation. However, how K48-linked ubiquitination is removed from MyD88 remains unknown. In the current study, we presented the identification of the K48-linked MyD88 deubiquitinase and how it modulates the stability of MyD88. Our results demonstrated for the first time how the TLR-NF-κB innate immune signaling pathway is manipulated by the deubiquitination of its central adaptor MyD88.

In general, PTM of MyD88 plays vital roles in its function and in the regulation of innate immune signaling homeostasis. A previous study has uncovered that MyD88 is post-transcriptionally regulated by tyrosine phosphorylation (14). SYK is a critical kinase that promotes MyD88-dependent signaling by phosphorylating MyD88; this effect can be inhibited by SHP1-mediated SYK inactivation (14). K48-linked ubiquitination of MyD88 by E3 ligases also has critical impacts on MyD88-dependent TLR signals. The E3 ligases known to be involved in ubiquitination and degradation of MyD88 include Cbl-b, Nrdp, Smurf1 and Smurf2, and the recently-identified SPOP complex (6–10).Two deubiquitinases, CYLD and OTUD4, have also been shown to remove K63-linked ubiquitination from MyD88; however, no K48-linked deubiquitinase of MyD88 has yet been found. In the present study, using chicken as a model animal, we observed that USP7 serves as a bona fide deubiquitinase of MyD88 and thus regulates the TLR-NF-κB signaling pathway. Specifically, chUSP7 interacts and co-localizes with chMyD88, removes polyubiquitination modification of MyD88, and increases its protein stability. As MyD88 protein abundance is tightly associated with TLR signaling activity, we observed cellular manipulation of *chUSP7* expression to positively regulate the expression of proinflammatory cytokines such as *IL-1β* and *IL-8*. Notably, uncontrolled TLR signaling is involved in the pathogenesis of a variety of autoimmune diseases. As MyD88 is the central adaptor of the innate signaling pathway, its deubiquitination by USP7 adds a new dimension to the complex network regulating TLR-mediated innate immune responses.

Emerging evidence suggests that USP7 controls the stability of a diverse set of substrates and is involved in a range of cellular processes, including tumorigenesis (15), adipocyte differentiation (16), amyotrophic lateral sclerosis (ALS) pathogenesis (17), inflammasome activation (18), and antiviral signaling. In addition, a physical interaction between the ubiquitin-like domain 2 of USP7 and the p65 subunit of NF-κB has been reported, revealing association of USP7 with the NF-κB pathway (19). USP7 inhibition has been determined to inhibit the expression of LPS-induced NF-κB target genes such as *Tnf-α*, *IL-6*, *IL-1β*, and *IL-12β* (19). Consistent with the above findings, our results demonstrated a positive role for USP7 in regulation of the LPS-induced TLR-NF-κB pathway, though we found USP7 to target the adaptor MyD88, which is upstream of p65. Taken together, all of the above findings indicate that USP7 regulates TLR-NF-κB signaling in multiple respects, targeting more than one member of the pathway. In the present study, we also revealed a critical impact of USP7 on mouse susceptibility to *Salmonella* infection. As expected, mice in which USP7 activity was inhibited by P5091 showed dramatically increased susceptibility to *Salmonella typhimurium*. Moreover, spleen expression of proinflammatory cytokines was significantly inhibited in P5091-treated mice, a finding highly consistent with what we observed at the cellular level.

In conclusion, we demonstrated that USP7-promoted K48-linked deubiquitination and stability of MyD88 is a novel mechanism that positively regulates MyD88-dependent proinflammatory signaling.

## Data availability statement

The original contributions presented in the study are included in the article/**Supplementary Material**. Further inquiries can be directed to the corresponding authors.

## Ethics statement

Animal care was performed in accordance with the regulations in the Guide for the Care and Use of Laboratory Animals issued by the Ministry of Science and Technology of the People’s Republic of China. The animal experiments were reviewed and approved by the Animal Ethics Committee of the Institute of Animal Sciences, Chinese Academy of Agricultural Sciences (Approval Number: IAS2018-8).

## Author contributions

QL, FW, and NZ conceived and designed the study. QL and FW wrote the manuscript. QL, GPZ, and JW provided funds. QL, NZ and FW performed the experiments and analyzed the data. GMZ, QW, YHL, QW, MSE and MZ helped in performing experiments.

## Funding

The work was funded by the National Key Research and Development Program (2018YFE0128000 to JW), Hainan Yazhou Bay Seed Lab (B21HJ0202 to QL), and the National Natural Science Foundation of China (320727008 to GZ).

## Conflict of interest

The authors declare that the research was conducted in the absence of any commercial or financial relationships that could be construed as a potential conflict of interest.

## Publisher’s note

All claims expressed in this article are solely those of the authors and do not necessarily represent those of their affiliated organizations, or those of the publisher, the editors and the reviewers. Any product that may be evaluated in this article, or claim that may be made by its manufacturer, is not guaranteed or endorsed by the publisher.

## Supplementary material

Supplementary Figure 1 Knockdown efficiency of different siRNAs designed to target chUSP7. The mRNA levels of *chUSP7* were examined by qRT-PCR.

Supplementary Figure 2 Expression of *chUSP7* and *chMyD88* mRNA in DF1 cells with overexpressed **(A)** or inhibited chUSP7 **(B)**.

Supplementary Figure 3 Expression of *IL-1β*, *IFN-α*, and *IL-6* in the spleen of P5091-treated mice challenged with Salmonella typhimurium for 6 h (n = 5 per group).
