# Supplementary figures and images for: USP7 Promotes deubiquitination and stabilization of MyD88 to enhance immune responses
Source: Front Immunol. 2022 Aug 12;13:900243. doi: 10.3389/fimmu.2022.900243 (PMC9412818; doi:10.3389/fimmu.2022.900243)

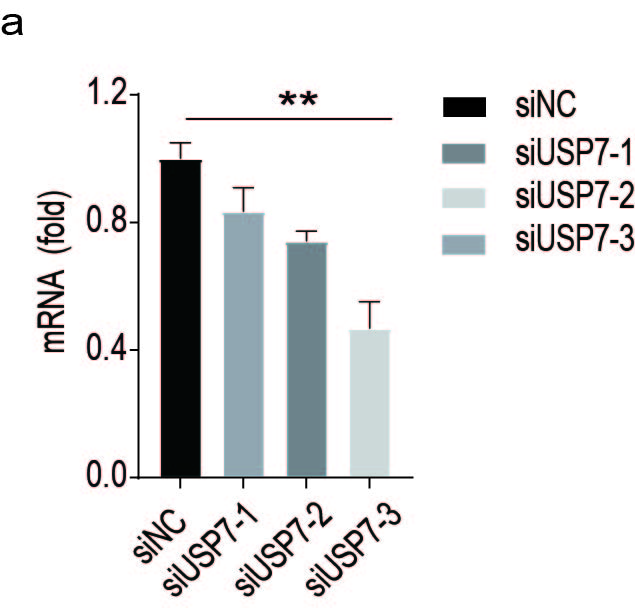

Supplement: Supplementary Figure 1 — Knockdown efficiency of different siRNAs designed to target chUSP7. The mRNA levels of chUSP7 were examined by qRT-PCR. [file Image_1.jpeg]

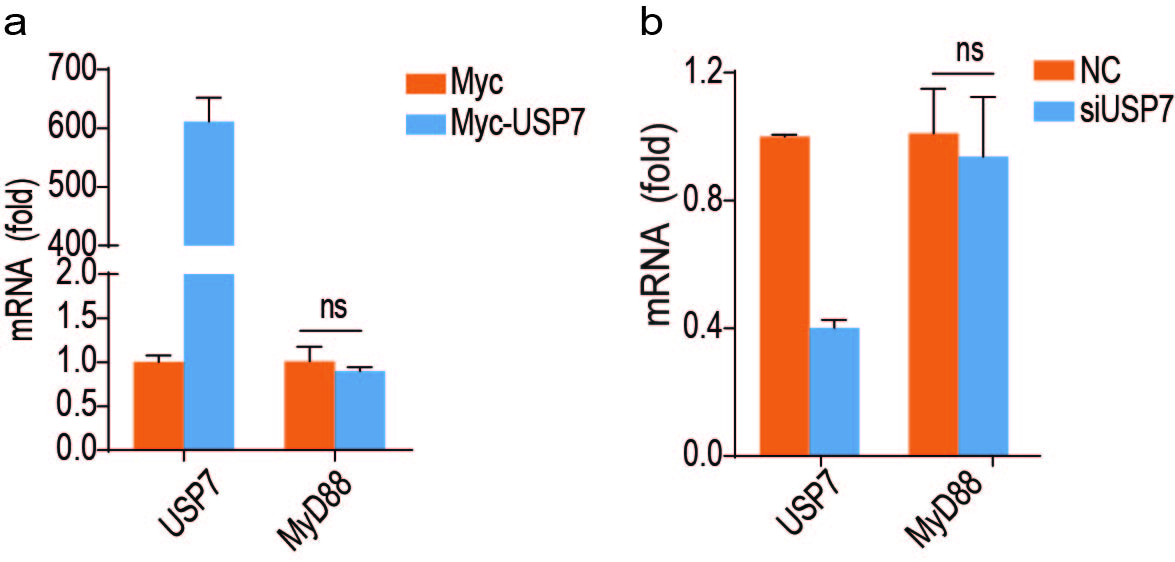

Supplement: Supplementary Figure 2 — Expression of chUSP7 and chMyD88 mRNA in DF1 cells with overexpressed (A) or inhibited chUSP7 (B). [file Image_2.jpeg]

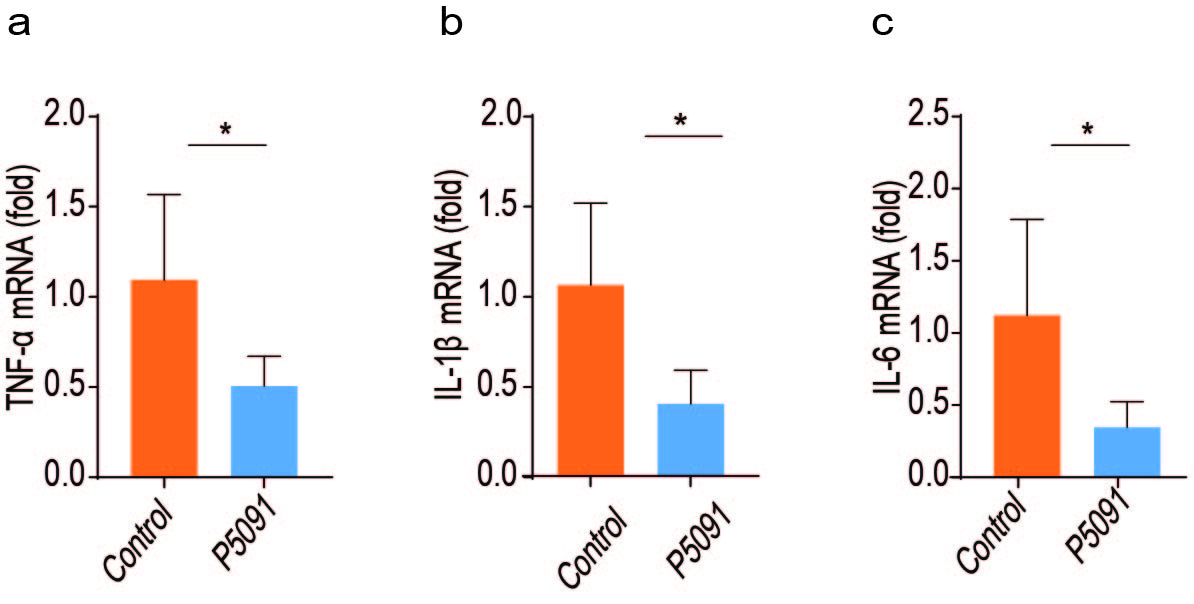

Supplement: Supplementary Figure 3 — Expression of IL-1β, IFN-α, and IL-6 in the spleen of P5091-treated mice challenged with Salmonella typhimurium for 6 h (n = 5 per group). [file Image_3.jpeg]
